# Development Strategies for Herbal Products Reducing the Influence of Natural Variance in Dry Mass on Tableting Properties and Tablet Characteristics

**DOI:** 10.3390/pharmaceutics4040501

**Published:** 2012-10-08

**Authors:** Ylber Qusaj, Andreas Leng, Firas Alshihabi, Blerim Krasniqi, Thierry Vandamme

**Affiliations:** 1 Laboratoire de Conception et d´Application de Molécules Bioactives (UMR-7199), Faculté de Pharmacie, UdS-CNRS, 67400 Illkirch-Graffenstaden, France; Email: alshihabi@unistra.fr (F.A.); vandamme@unistra.fr (T.V.); 2 Research and Development Pharma, Bioforce AG, 9325 Roggwil (TG), Switzerland; Email: a.leng@bioforce.ch (A.L.); blerim.krasniqi@hepart.ch (B.K.)

**Keywords:** herbal material, liquid fresh plant extract, tableting properties, Echinacea purpurea, wet granulation, solid dosage form

## Abstract

One “Quality by Design” approach is the focus on the variability of the properties of the active substance. This is crucially important for active substances that are obtained from natural resources such as herbal plant material and extracts. In this paper, we present various strategies for the development of herbal products especially taking into account the natural batch-to-batch variability (mainly of the dry mass) of tablets that contain a fixed amount of tincture. The following steps in the development have been evaluated for the outcome of the physico-chemical properties of the resulting tablets and intermediates: concentration of the tincture extracted from Echinacea fresh plant, loading of the concentrate onto an inert carrier, the respective wet granulation and drying step, including milling, and the adjuvant excipients for the tablet compression step. The responses that were investigated are the mean particle size of the dried and milled granulates, compaction properties and disintegration time of the tablets. Increased particle size showed a significant increase of the disintegration time and a decrease of the compaction properties. In addition, our results showed that the particle size has a great dependency on the ratio of liquid to carrier during the wet granulation process. Thus, the variability of the respective parameters tested was influenced by the performed strategies, which is how the tincture correlated to its dry mass and the relation of the amount of carrier used. In order to optimize these parameters, a strategy considering the above-mentioned points has to be chosen.

## 1. Introduction

In the “Quality by Design” approach in formulation development, it is important to take into consideration the properties of the active substances and their impact on the final dosage form. This is especially important for attributes, which show a high degree of variability and which can not be controlled by the production process itself.

Stemming from naturally grown products, herbal remedies are the best examples of these kinds of natural variations. These variations are a complex result of several factors such as the soil condition, climatic conditions, the vegetation time and the harvesting and maceration processes.

Within the last few years, herbal remedies have become more and more popular as alternatives to conventional pharmaceuticals. This is because of their expansive use in several applications often with minor side effects compared to chemical pharmaceutics [1].

The most common dosage forms of herbal preparations are liquids derived from macerations, infusions and decoctions [2], with the associated problems of large dose volumes, impractical packaging and poor stability [3]. Solid preparations or tablets on the other hand often have higher stability and are easier to standardize which adds to an increase in their therapeutic acceptance, efficacy and product value [4]. Tablets are simple and convenient dosage forms that enable accurate tamper proof doses to be delivered.

The formulation of herbal medicines particularly when using fresh plants for robust tablet forms is challenging due to the inherent poor tableting properties of most herbal extracts or powdered plant parts. In addition, larger amounts of the liquid used for extraction with limited information on relevant physico-technical properties of these crude drugs in relation to the commonly used excipients can be a problem [5]. Depending on the natural variation of the dry mass of the tincture, the tablets will contain changing amounts of dry extract, hence the need to sometimes standardize to a fixed amount of tincture per tablet. Dry mass of the herbal extract is the material consisting only of the components present in the original plant or material extracted during the extraction process, excluding any excipients or other added substances [6]. The amount of dry extract has either a higher or lower impact on the process ability during tableting and on the properties of the tablets.

Standardization of the concentrate is important in diminishing the variability of the dry mass in the tincture. It allows relation of the volume to its constituents and reduces variability in its properties. The degree of freedom is reduced depending on the amount of variables that have been standardized. In the present study, it was important to keep the ratio of ethanol / water in the liquid phase constant, so that only the concentration could be varied or fixed. A focus on two types of standardization of dry mass inside the concentrate was therefore studied: a fixed concentration standardization and a fixed volume standardization.

Here we present various strategies for the development of a herbal drug tablet with the equivalence of the tincture taking into account the natural variability (mainly of the dry mass).

As a model for a herbal remedy we are using a tablet made from Echinacea purpurea tincture. Echinacea purpurea is a very common herbal plant and its preparations represent the most commonly used herbal immune modulator, with antiviral, antibacterial effects predominantly used against the common cold [7].

Echinacea purpurea tincture is used as a starting substance for the tablets. It is then concentrated to form a soft extract, which is loaded onto a different carrier to form granulates. After drying, milling and mixing with further excipients, the mixture is pressed.

In this paper, we focus on the variability of the dry mass of the tincture and its influence on the compaction properties of the mixture and the disintegration time of the tablet. Furthermore, the relation between these parameters and the granule size will be discussed.

## 2. Materials and Methods

### 2.1. Materials

Echinacea purpurea tincture is provided by Bioforce AG, Roggwil, Switzerland. β-cyclodextrin (β-CD); (Wacker Chemie GmbH, Germany); Microcrystalline Cellulose (MCC) (JRS Pharma, Rosenberg, Germany); Lactose monohydrate (Domo, Zwolle, Netherlands) and magnesium stearate (Peter Greven, Bad Münsterreifel, Germany), all of which were pharmaceutical grade.

### 2.2. Methods

#### 2.2.1. Preparation of Liquid *Echinacea Purpurea* Extract

Evaporation of *Echinacea purpurea* fresh plant tincture and final adjustment of the liquid amount: The tincture of *Echinacea* is a hydro-alcoholic extract (Ethanol content 65%) made from the freshly harvested herbs and roots of *Echinacea purpurea*. The solvent of *Echinacea* tincture was evaporated under reduced pressure at 45 °C. The extract was dried in a vacuum oven at 45 °C to constant weight. Subsequently, the water and ethanol were added to gain the needed experimental conditions that represent the soft extract in our products within its respective variations which are listed in Table 1.

#### 2.2.2. Preparation of the Granulate

A granulate was prepared in a lab size high shear mixer (Kenwood) by blending beta-cyclodextrin (β-CD) with the concentrate of *Echinacea purpurea* extract and subsequently mixing this into the carrier (microcrystalline cellulose (MCC)), according to the design of each of the specific experiments (see Table 1). β-CD (5% in formulation) was used to mask the taste of the *Echinacea* tincture within the tablets. The wet granulate was sieved through a 2 mm sieve and dried overnight (14 h) at 45 °C in an oven. In order to get homogenious particle size distribution and to avoid variation in the drug content during the tableting process, the dried granulate was sieved by a rotary sieving machine (MG205, Frewitt, Switzerland, 0.63 mm sieve). The particle size was evaluated as described under granules characteristics.

**Table 1:** Parameters of the experimental setting for the development of an herbal solid dosage form.

	Dry mass in tincture (%)	Dry mass in concentrate (%)	Liquid amount (g)	Carrier amount (MCC) (g)
*Treatment 1:* *fixed concentration—fixed carrier standardisation*				
A	1.2	43.0	14.5	25.0
B	1.7	43.0	16.3	25.0
C	2.2	43.0	16.9	25.0
D	2.8	43.0	18.9	25.0
*Treatment 2:* *fixed concentration—fixed carrier with compensation standardisation*				
E	1.3	43.0	14.4	21.0
F	1.5	43.0	15.4	25.0
G	2.0	43.0	15.8	33.0
H	2.5	43.0	16.9	42.0
*Treatment 3* *:* *fixed volume—fixed carrier standardisation*				
I	1.3	32.0	15.0	25.0
J	1.5	37.5	14.2	25.0
K	2.0	50.0	12.2	25.0
L	2.5	62.5	10.2	25.0
*Treatment 4* *:* *fixed volume—fixed liquid to carrier ratio standardisation*				
M	1.3	32.0	16.4	27.3
N	1.5	37.5	15.3	25.5
O	2.0	50.0	14.7	24.5
P	2.5	62.5	13.5	22.5

#### 2.2.3. Preparation of Tablets

The dried and sieved granulate was blended in a turbula mixer (Turbula, Basel, Switzerland) with adequate excipients (q.s.), and magnesium stearate (0.5 g) resulting in 100.0 g of mixture, which was ready for pressing. Tablets (250 mg) were pressed by an eccentric tablet press (Fette Exacta 21, Fette GmbH, Schwarzenbek, Germany) equipped with six punches (8 mm round, oval). The speed was set at 35 rpm and the compaction force was set to achieve a similar hardness for each formulation. For testing of the maximum breaking strength, each batch was compressed at the different compression force levels from 6.8–7.8 in the eccentric tablet press.

#### 2.2.4. Characterization of Granulate

##### 2.2.4.1. Particle Size Distribution

The particle size distribution was analyzed by a sieve analysis (Retsch, Germany). Sieving was performed using eight selected sieves ranging from 1000 µm to 90 µm. Particle size distribution was calculated as the ratio of the sieves cumulative weight and total sample mass. The mean particle size diameter (MPSD) (*d*) of each formulation was calculated with the following Equation 1:

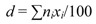

where *x_i_* is the arithmetic mean of the upper and lower limit of *i* sieve fraction (determined by the aperture size of the used sieve) and *n_i_* is the percentage of the particles weight of *i* sieve fraction [8].

The test was performed in triplicate and standard deviations were calculated. The significant correlation of the data was calculated within the software Minitab^®^ Version 16.1.0, (correlation coefficient (*r*) as well as the *p*-value (α)).

##### 2.2.4.2. Moisture Content

In order to check whether the residual moisture of the granulate can influence the compaction properties, the moisture content of the final mixture was measured. The moisture content of all the samples was monitored with the digital moisture analyzer (HR73, Mettler Toledo, Switzerland). The average of the three measurements were calculated.

##### 2.2.4.3. Bulk and Tapped Density

The bulk density was evaluated by determining the weight of 100 g (m) granulates in a graduate dry cylinder, 250 cm^3^. The apparent volume (*V*_0_) was read to the nearest graduated unit and the bulk density was calculated in grams per cubic centimeter [9]. The same sample used for the bulk density (100 g of granules) was used for the taped density. The 250 cm^3^ dry cylinder was secured to an automatic tapper (Stav 2003, Stampfvolumeter, Switzerland). The final tapped density was calculated by reading the volume after (1250) tapping and using Equation 2:

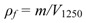


The average of the three above-mention measurements was presented and standard errors of the mean were calculated.

#### 2.2.5. Tablets Characterization

##### 2.2.5.1. Disintegration Time

Disintegration time is the time required for a breakdown of the tablet into smaller particles that pass through the 2 mm screen completely. The procedure using the basket rack assembly with a disk was carried out in the disintegration tester (ZT72, Erweka, Heusenstamm, Germany). Distilled water maintained at 37 ± 0.5 °C was used as the test medium. Six tablets from each formulation were tested and the mean of six tablets was recorded automatically in minutes and subsequently calculated into seconds. The standard errors of the mean were then calculated. The significant correlation of the data was calculated with the software Minitab^®^ Version 16.1.0, (correlation coefficient (*r*) as well as the *p*-value (α)).

##### 2.2.5.2. Compaction Properties

In order to evaluate the compaction properties of two different formulations, the maximum breaking strength was assessed. This represents the maximum breaking strength measured by a pneumatic tablet hardness tester (Pharmatron 4 M, Schleuniger, Solothurn, Switzerland) in a sequence of increasing compaction forces. This measurement was chosen because this value is independent of the applied forces and therefore does not need the measurement of the value of the forces applied. The mean of 6 tablets was recorded in newton (N) and the standard errors of the mean were calculated. The significant correlation of the data was calculated with the software Minitab^®^ Version 16.1.0, (correlation coefficient (*r*) as well as the *p*-value (α)).

### 2.3. Examining the Effect of Different Liquid to Carrier Ratios in the Granulation System

As a first step, the effect of the liquid amount and the carrier amount was examined on the mean particle size diameter (MPSD) of the granules. The accompanied effects on compression properties and disintegration time of the respective tablet were also assessed. For this reason, three amounts of concentrate and supportive liquids were added to three different amounts of MCC by performing a 3 × 3 factorial design (Table 2).

**Table 2:** Composition of the granules by using different amounts of microcrystalline cellulose (MCC).

Sample name	Liquid amount (g)	MCC (g)
1	8.7	12.5
2	8.7	17.5
3	8.7	25
4	12.1	12.5
5	12.1	17.5
6	12.1	25
7	15.4	12.5
8	15.4	17.5
9	15.4	25

### 2.4. Concentration Settings Depending on the Dry Mass

During the development of the formulation, several treatments were tested (Table 1). As a further step, the process of granulation had to be evaluated as there are several parameters that may have shown a significant influence. The principal aim was to standardize the rules of administration of the concentrate to the carrier and the supportive liquids. First of all, the treatments for the fixed concentration standardization concentrate had to be evaluated, followed by the treatments for fixed volume standardization concentrates. The compositions of the formulation (Treatments) are described in detail in Table 1.

#### 2.4.1. Fixed Concentration Standardization (FCS)

In this standardization, the concentration of the dry mass within the concentrate was fixed. As the dry mass concentration in the tincture was variable, the volume of the concentrate used for the production changed in accordance to the dry mass of the respective tincture when the product had to be adjusted according to the volume of the tincture. The fixed concentration standardization has the advantage that the physical properties of the concentrate prove similar. However, the chemical constituents can be different from one batch to another batch due to the different chemical constituents of the tincture.

##### 2.4.1.1. Treatment (1) Fixed Carrier

The simplest method was to add the required amount of concentrate to a fixed amount of carrier. The proportion between the concentrate and the carrier can vary (Table 1).

##### 2.4.1.2. Treatment (2) Fixed Carrier with Compensation

In order to reduce this variation the amount of carrier is based on the amount of concentrate in a fixed ratio. In order to compensate the loss of carrier in the formulation and to compensate different amounts in dry mass in the formulation, a certain amount of carrier had to be added afterwards in order to ensure the same amount of granulate was obtained. In addition, the water amount for the granulation step was standardized by adding more water to gain a fixed amount of liquid.

#### 2.4.2. Fixed Volume Standardization (FVS)

In the fixed volume standardization, the dry mass varies but the reduction of the volume is set by a fixed concentration factor. For example, if 1000 kg of the tincture is concentrated by a concentration factor of 20, it yields to 50 kg of concentrate. As the concentration of the dry mass is proportional to the dry mass of the tincture, the physical properties can differ from a good flowing soft extract to a more viscous mass. During development it is important to choose the correct concentration factor in order to get a concentrate that is suitable for further processing (not too thick and not too liquid under the given specification limits of the dry mass of the tincture). Since the same amount of the concentrate reflects the same amount of tincture, the amount of concentrate used for production is always the same.

##### 2.4.2.1. Treatment (3) Fixed carrier

Similarly to Treatment 1, the concentrate was added to a fixed amount of carrier. The amount of additional liquids was fixed. As the amount of concentrate was fixed due to the results of the standardization process, the fixed volume standardization, the amount of liquids only changed because of the differences in the concentration of native extract in the concentrate. No compensation for dry mass was done with the carrier. By adding different amounts of the major constituent (the filler) of the formulation which in our case was lactose, the variable amounts of the resulting granulates can be considered.

##### 2.4.2.2. Treatment (4) Fixed liquid to carrier ratio

As anticipated, the amount of liquid per carrier had a huge influence on the size of the granules, so a treatment was set up to fix the ratio of liquid to carrier. In addition, the amount of carrier was set so that the resulting amount of granulate was fixed; therefore, no compensation in a later step was necessary.

**Figure 1:**
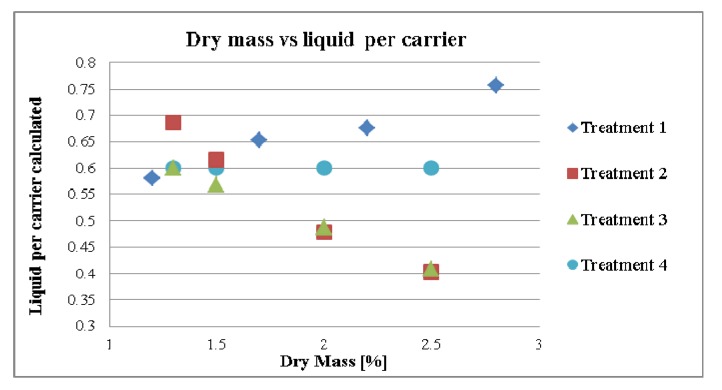
The ratio of liquid per carrier calculated in relation to the dry mass.

Figure 1 shows a chart of the calculated liquid per carrier ratio in relation to the dry mass of the tincture as a result of concentration with the addition of further liquid. This additional liquid is used for cleaning and rinsing, as well as to keep the concentration of total water constant (Treatments 1 and 2).

## 3. Results and Discussion

### 3.1. Influence of the Liquid per MCC on the Compaction Properties and Disintegration Time

**Figure 2:**
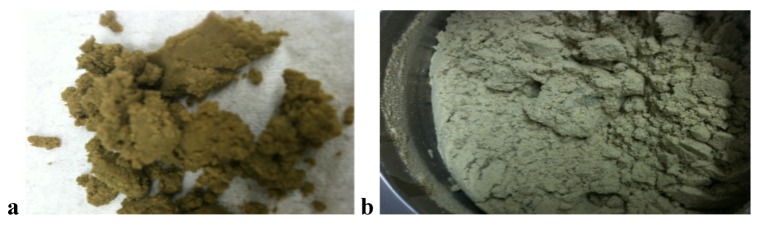
(**a**) Granulate containing a higher liquid amount in the formulation. (**b**) Granulate containing a lower liquid amount in the formulation.

**Figure 3:**
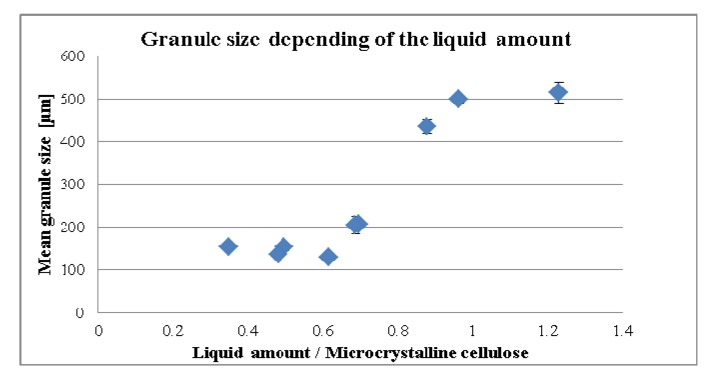
Mean granule size depending on the liquid amount.

During the experiments, it was observed that the amount of the total liquids per carrier had an influence on the physical character of the granulate. High volumes of liquids in comparison with the amount of carrier led to “wet massing granules” (Figure 2a) whereas a smaller amount of water only resulted in “dried” granules (Figure 2b). This outcome was quantified by measuring the resulting MPSD (Figure 3). In this figure the ratio of liquid to carrier was plotted against MPSD results resulting in a sigmoidal shape. 

It can be described as having a kind of plateau at 150 µm which started at about 0.6 points increasing to about 1 point when there is a transition into a second plateau at about 500 µm. 

One should consider that the lower plateau can be related to the particle size of the carrier, which was not well granulated since the granulation at the lower liquid per carrier had not taken place. In contrast, the upper plateau obviously depended on the sieve size the granulate was treated to with a given sieve size of 500 µm. Increasing the liquid amount per carrier in the formulation increased the granule size significantly (*p*-value = 0.001, *r* = 0.912). Evolution of mean granule size during liquid addition was mentioned by Benali *et al*. [10].

**Figure 4:**
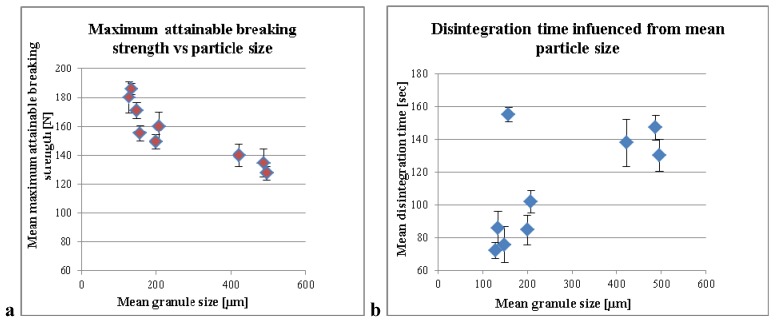
(**a**) Maximum breaking strength of the tablets compared to different mean particle size; (**b**) Disintegration time of the tablets compared to different mean particle size.

Furthermore, mean granule size diameter also showed an influence on the maximum breaking strength of the tablets (Figure 4a). By increasing the mean granule size, a decrease of maximum attainable breaking strength of the tablets was caused. The significant correlation of the data was calculated (correlation coefficient (*r*) as well as the *p*-value (α)). The data was accepted as a significant correlation, if the significance level was below *α* = 0.05. A significant correlation of MPSD to the maximum attainable breaking strength was found (*p*-value = 0.002, *r* = −0.884).

The diagram depicts that the disintegration time of the tablets was prolonged by increasing the mean granule size of the granules (Figure 4b). However, it only shows a significant correlation between MPSD and disintegration time, if experiment Nr. 2 (Table 1) of the calculation is excluded (*p*-value = 0.000, *r* = 0.954). The disintegration value of sample 2 appeared to be a little odd, since it had the same amount of liquid as in samples 1 and 3 with the amount of MCC being almost in the middle of them both. While both show disintegration times of 107 and 88 s, sample 2 shows disintegration time of 155 s. By including experiment 2 in the calculation, the significance diminished (*p*-value = 0.060, *r* = 0.647).

In the mentioned study MPSD seems to be the main factor that influenced the compression properties (and more than likely the disintegration time) of the tablets. The effect of the granule size in the disintegration time and capping in the compressing of the tablets was investigated by Forlano *et al*. [11]. From these experiments, we demonstrated that the liquid amount per carrier was the key factor for the next step of the development of the tablet formulation.

Increasing the liquid amount in the formulation required an increase of the carrier in order to obtain the same granule size. Small granule size (100–200 µm) was preferred in order to achieve a fast disintegration time and maximum compaction properties during the development of the tablets. Therefore in the granulation process, the liquid amount per carrier should be accomplished below a liquid to carrier ratio of 0.7 (Figure 3) for the required properties of the tablets.

### 3.2. Standardization Strategies

Standardization strategies for the extraction process and reduction of the liquid amount of herbal extracts are very important in order to reduce the variability of the drug content in the dry mass of Echinacea maceration. It is advisable to start with standardization as early as possible. Therefore, the mode and date of harvesting [12], the geological properties of the fields and the parts of the plant were determined. However, despite the attempts to control all of these factors, some environmental conditions such as the weather, sunshine, duration of seasons, *etc*. cannot be controlled. Owing to these conditions unavoidable variations from batch to batch occur which is reflected in the variable amount of ethanolic/aqueous soluble substances. These components that form the “native extract” can be determined as the amount of dry mass in the tincture. The chemical composition of the Echinacea extract is complex, and it appears that the most pharmacologically active compounds are the alkylamides, phenylpropanoids and polysaccharides [13]. Due to its complexity it is impossible and impracticable to assess the effect of each component on the tableting properties. They have to be handled as a whole.

#### 3.2.1. Effect of Dry Mass of the Tincture on Granule size (MPSD) for Different Treatment Strategies

**Figure 5:**
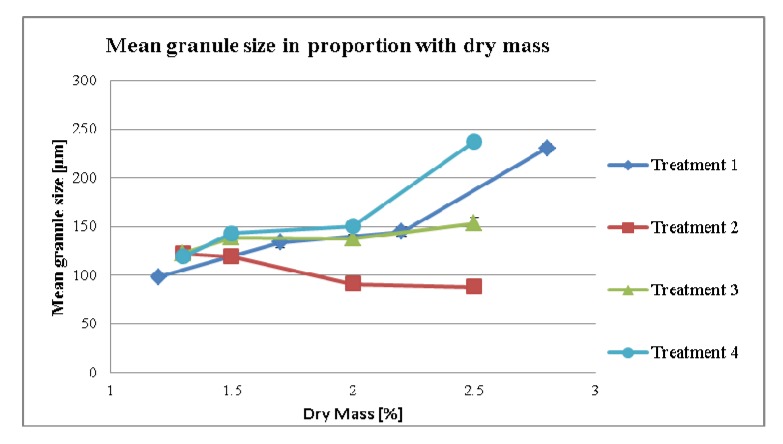
Mean granule size to dry mass of tincture.

MPSD of granulates are shown in Figure 5. It was expected that the changes in the dry mass of the tincture would lead to changes in the granule size according to its liquid per carrier value as seen in Figure 1. Thus, by increasing the liquid amounts in proportion to carrier in the formulation, the MPSD in granulate (treatments 1 *p*-value = 0.048, *r* = 0.952 and treatment 2 *p*-value = 0.058, *r* = −0.942) increased. As it can be seen there is a difference in treatment 2 to treatment 1 as the liquid/carrier levels go down in treatment 2 whereas the go up in treatment 1. As with increasing the dry mass in the tincture the MPSD increases in treatment 1 but decreases in treatment 2 it seems that the increase in liquid/carrier is more important than the % of dry mass, which also leads to a higher content of native extract within the granulate. This phenomena was also described by Ohno *et al*. [14]. He noted that by increasing the water amount and the kneading time the particle diameter increases. On the other hand in treatment 3 the MPSD increased slightly although the calculated liquid per carrier decreased (*p*-value = 0.104, *r* = 0.896). In this treatment, another factor seemed to influence the MPSD. It could be that the dry mass in treatment 3 probably superimposed the reduction of the MPSD. In addition, the liquid per carrier value was below 0.7. Therefore, the influence of the liquid per carrier was considered present but very low.

In treatment 4 (Figure 5) all the samples had the same liquid per carrier ratio. Interestingly, the MPSD increased with an increasing dry mass (*p*-value = 0.070, *r* = 0.930). As the major difference is the amount of the native extract per granules, it can be concluded that the dry mass content of the tincture enhances the resulting granule size. As a result we see that in treatment 3 even with the changes in dry mass, only a very slight increase in the MPSD is seen.

**Figure 6:**
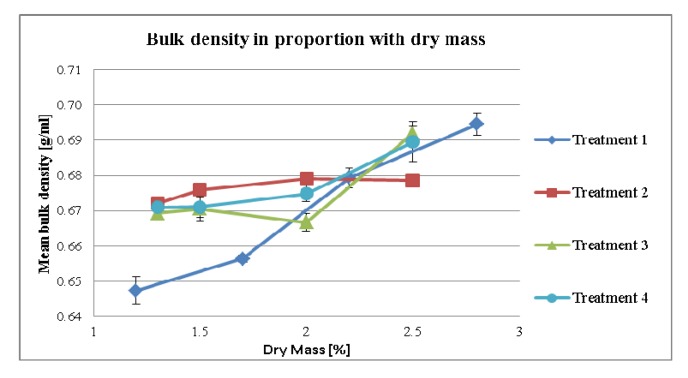
Bulk density of mixture depending on dry mass of tincture.

**Figure 7:**
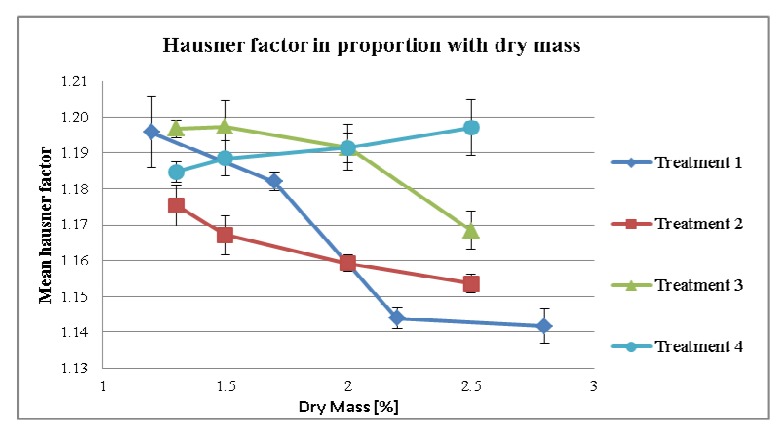
Flowability of granulate depending on dry mass of tincture.

As expected, by increasing MPSD an increased bulk density of the final mixture occurs. This phenomenon was observed throughout the treatments, which is prominent for treatments 1 and 4 (Figure 6). In addition, the flowability was increased as indicated by the reduced Hausner factor (Figure 7).

#### 3.2.2. Effect of Dry Mass of the Tincture on Compaction Properties for Different Treatment Strategies

To measure the maximum breaking strength of the tablets, the compaction force was increased continuously (but not necessarily linearly) until the highest level of breaking strength was reached. The reasons for this can be explained by the fact that higher compression forces induced “capping” during the tableting process or that the porosity of the tablet was too low for further compaction.

**Figure 8:**
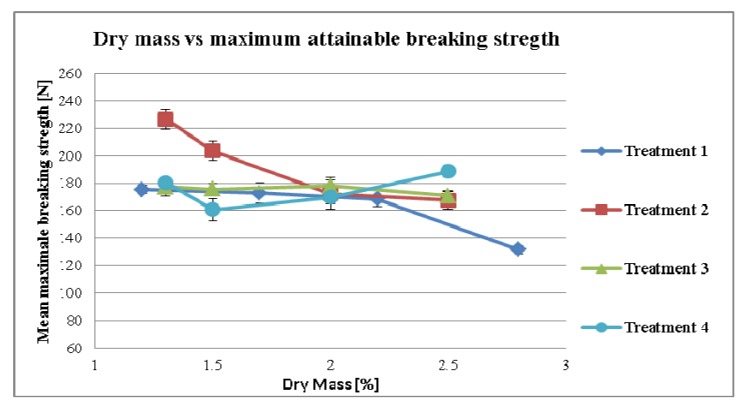
Maximum attainable breaking strength depending on dry mass of tincture.

The maximum attainable breaking strength of the tablet recorded from the formulation is presented in Figure 8.

Figure 8 demonstrates that the effect of the mean granule size was not as pronounced as such in Figure 4a. The effect of the dry mass was observed in treatment 1 as a reduction of the maximum attainable breaking strength at point 2.8%. In the first three points of treatment 1 we see that the values describes an almost horizontal line (*p*-value = 0.122, *r* = −0.878). The point 2.8 seems substantially lower. However, given that Figure 5 we see that the MPSD of the 2.8% sample is also remarkably higher than the first three samples this could explain why the reduction in hardness is evident only in the 2.8% sample.

There was no effect of the dry mass in treatments 3 (*p*-value = 0.285, *r* = −0.715) and treatment 4 (*p*-value = 0.491, *r* = 0.509) on the maximum attainable breaking strength. However, in treatment 2 additional extragranular MCC seems to show effects on the compaction properties (*p*-value = 0.065, *r* = −0.935). The extragranular MCC was added in the formulation to compensate the variabilities of the dry mass related to the Echinacea tincture. This additional MCC could increase the compaction properties of the granulate [14].

It is unavoidable that the different amount of dry mass leads to different amounts of granulates. Three different approaches were used to solve this issue. First of all, in doing so we could compensate the loss or the excess with the filling material of the tablet (in our case lactose). This postponed the compensation step to a later processing step. Therefore mixing different batches could have proven difficult (Treatments 1 and 3). Secondly, we could use the carrier as a compensation material, adding the missing amount to the dried granulate after the milling step (Treatment 2). Thirdly, we calculated the amount of carrier according to the amount of dry mass in the tincture respectively with the amount of dry mass in the concentrate (Treatment 4). Since the amount of “free” MCC was changed in treatment 2 to higher proportions, it showed the highest variations in maximum attainable breaking strength. If the goal was a reduction of variations on the tableting properties coming from the variation of the tincture, compensation with additional MCC might not be a good idea, as it induced additional variations.

One might also ask whether a different moisture content of the final mixture could contribute to the effect. As we did not observe any differences in the moisture content within each treatment or between the treatments (Table 3), it is unlikely that it can be made responsible for this. 

**Table 3:** Moisture content for different treatment strategies.

Dry Mass (%)	Treatment 1	Treatment 2	Treatment 3	Treatment 4
1.2	3.1			
1.7	3.4			
2.2	2.72			
2.8	3.96			
1.3		2.92	2.96	2.77
1.5		2.96	3.04	2.48
2		3.13	2.79	2.52
2.5		2.27	3.36	3.74

#### 3.2.3. Effect of Dry Mass of the Tincture on Disintegration Time for Different Treatment Strategies

**Figure 9:**
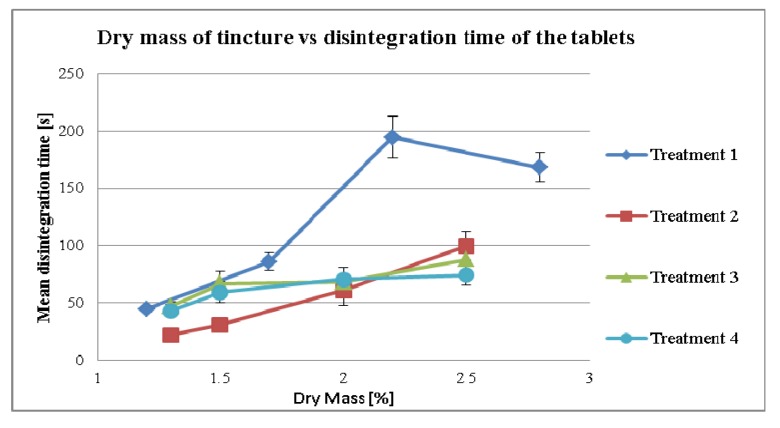
Disintegration time of different treatments influenced by the dry mass of tincture.

In Figure 9, the disintegration time of the different treatments have been described. There was an overall effect of dry mass on disintegration time (*p* = 0.01) and on treatment (*p* < 0.05) analyzed by ANOVA. 

The effect of the higher amount of dry mass in disintegration time was observed more in Treatment 1 (*p*-value = 0.029, *r* = 0.916). All treatments were processed similar. However Treatment 1 yielded into slightly higher breaking strength values than the other treatments although the compression forces levels were equal. Higher breaking strength often is associated with increased disintegration time. This might explain why Treatment 1 could have prolonged disintegration times. As the 2.2% sample does not fit to the line in Treatment 1 we assume that this would be some kind of sampling error. Retesting shows that the values of the 2.2% sample are below the samples of 2.8%. However, since there is some kind of increase in disintegration time over time, these values cannot be used for showing them to this investigation. As the 2.8% sample lies in somehow line of the first two samples we consider it value as correct. However it is interesting to observe, that with increasing dry mass all treatments showed almost linear correlation. Treatment 2 showed a faster disintegration time, caused by the addition of the extra granular MCC. The influence of the dry mass and additional MCC was significantly correlated (*p*-value = 0.005, *r* = 0.995). In Treatments 3 and 4 the effects on the higher amount of dry mass, showed no significant correlation in the disintegration time, Treatment 3 (*p*-value = 0.078, *r* = 0.922), treatment 4 (*p*-value = 0.093, *r* = 0.907).

A higher amount of the dry mass negatively influenced the disintegration time of the Echinacea tablets. In the literature, the effect of the herbal dry extracts to increase tablet hardness and to prolong disintegration time is usually described [15] due to their hygroscopic nature. Moreover, additional MCC [5] in the tablet can induce a water uptake, swelling and a quick rupture of the compact. Whereas, additional “free” MCC as a compensation material could reduce the disintegration time, but could be also a source for variability of the disintegration time similar to the one described above.

In the first experiments we demonstrated that the amount of liquid to the amount of carrier as having a strong influence on the MPSD. Furthermore, this was accompanied by lower compaction properties possibly due to the increase of the disintegration time.

In terms of strategies, which should erase the effects of the natural variation in dry mass, four different strategies have been presented. Two strategies demonstrated a fixed concentration standardization and two strategies a fixed volume standardization concentrate. In each group the concentrate is added to a fixed amount of carrier with one with some “useful” compensatory modifications.

Here, the MPSD of the granulate is used as a parameter to determine the character of the granulates and the granulation process. Depending on the amount of the liquid used per carrier it shows a sigmoidal shaped curve with two plateaus.

In the context of the development, it is recommended to work in either the lower or the upper plateau in order to reduce the variability.

In addition, the standardization of the concentrates and the granulation are important. With different concentrations of the dry mass, the liquid per carrier ratio, the total amount of granulate, the quality and quantity of the excipient for compensation, the resulting MPSD can change. These factors can influence compression properties and disintegration time of the tablets massively together with the already changing amount of dry mass.

Standardizations are also increasingly prone to errors. The more parameters needed to be standardized and the more parameters which need to be considered, the more susceptible the system. Therefore, an easy and less complicated standardization should be preferred.

In this specific study a simple standardization (Treatment 3) well performed can easily be demonstrated in terms of eliminating the variations. In a range of dry mass 1.3%–2.5%, it showed the lowest differences in the MPSD; small differences in maximum breaking strength and together with Treatment 4 the lowest differences in disintegration time.

## 4. Conclusion

To summarize, it was observed that in developing herbal drug tablets equivalent to the tincture, standardization of concentration should be emphasized. The standardization of the concentration and granulation has a strong influence on how a present variation may affect the final result. Basic test models can give a first impression of how a system can work. However, only working through the various treatment models can show whether these attempts will work in reality.
